# Neural network-based prognostic predictive tool for gastric cardiac cancer: the worldwide retrospective study

**DOI:** 10.1186/s13040-023-00335-z

**Published:** 2023-07-18

**Authors:** Wei Li, Minghang Zhang, Siyu Cai, Liangliang Wu, Chao Li, Yuqi He, Guibin Yang, Jinghui Wang, Yuanming Pan

**Affiliations:** 1 Cancer Research Center, Beijing Chest Hospital, Capital Medical University, Beijing Tuberculosis and Thoracic Tumor Research Institute, No.9 Beiguan Street, Tongzhou District, Beijing, 101149 China; 2 Dermatology Department, General Hospital of Western Theater Command, No.270 Tianhui Road, Chengdu, 610083 Sichuan Province China; 3 Institute of Oncology, Senior Department of Oncology, the First Medical Center of Chinese CLA General Hospital, No.28 Fuxing Road, Haidian District, Beijing, 100853 China; 4 Department of Gastroenterology, Peking University Aerospace School of Clinical Medicine, No.15 Yuquan Road, Haidian District, Beijing, 100049 China; 5 Department of Gastroenterology, Beijing Chest Hospital, Capital Medical University, Beijing Tuberculosis and Thoracic Tumor Research Institute, No.9 Beiguan Street, Tongzhou District, Beijing, 101149 China

**Keywords:** Neural network, Deep learning, Gastric cardiac cancer, Survival prediction

## Abstract

**Backgrounds:**

The incidence of gastric cardiac cancer (GCC) has obviously increased recently with poor prognosis. It’s necessary to compare GCC prognosis with other gastric sites carcinoma and set up an effective prognostic model based on a neural network to predict the survival of GCC patients.

**Methods:**

In the population-based cohort study, we first enrolled the clinical features from the Surveillance, Epidemiology and End Results (SEER) data (*n* = 31,397) as well as the public Chinese data from different hospitals (*n* = 1049). Then according to the diagnostic time, the SEER data were then divided into two cohorts, the train cohort (patients were diagnosed as GCC in 2010–2014, *n* = 4414) and the test cohort (diagnosed in 2015, *n* = 957). Age, sex, pathology, tumor, node, and metastasis (TNM) stage, tumor size, surgery or not, radiotherapy or not, chemotherapy or not and history of malignancy were chosen as the predictive clinical features. The train cohort was utilized to conduct the neural network-based prognostic predictive model which validated by itself and the test cohort. Area under the receiver operating characteristics curve (AUC) was used to evaluate model performance.

**Results:**

The prognosis of GCC patients in SEER database was worse than that of non GCC (NGCC) patients, while it was not worse in the Chinese data. The total of 5371 patients were used to conduct the model, following inclusion and exclusion criteria. Neural network-based prognostic predictive model had a satisfactory performance for GCC overall survival (OS) prediction, which owned 0.7431 AUC in the train cohort (95% confidence intervals, CI, 0.7423–0.7439) and 0.7419 in the test cohort (95% CI, 0.7411–0.7428).

**Conclusions:**

GCC patients indeed have different survival time compared with non GCC patients. And the neural network-based prognostic predictive tool developed in this study is a novel and promising software for the clinical outcome analysis of GCC patients.

**Supplementary Information:**

The online version contains supplementary material available at 10.1186/s13040-023-00335-z.

## Background

**Fig. 1:**
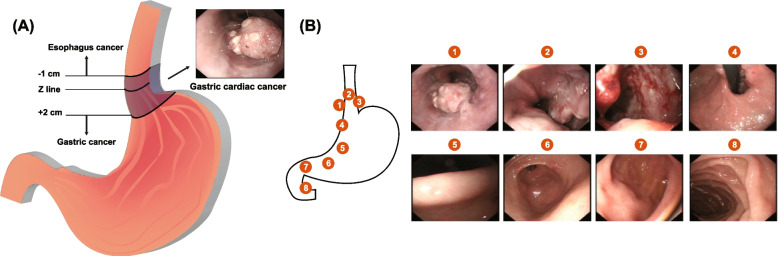
Schematic of gastric cardiac cancer (GCC). **A** Location of GCC. **B** White-light endoscopy image of GCC

Gastric cancer (GC), as the GLOBOCAN 2020 reported, is the fifth most common cancer and the fourth primary cause of tumor-related death globally with over 1.08 million new cases and nearly 0.77 million deaths (about one out of every 13 deaths died of gastric cancer) [1]. The incidence of males is more than twice that of females. Gastric cancer can be anatomically divided into two categories: gastric cardiac cancer (GCC) and other sites of GC (non-gastric cardiac cancer, NGCC). Generally speaking, the midpoint of the GCC is between 1 cm proximal and 2 cm distal from the gastroesophageal junction with endoscopic image acquisition of different parts of stomach [2] (Fig. 1). In the past half century, the incidence of NGCC has decreased, replaced by the high incidence of GCC worldwide [3, 4]. Due to the rapid progression and metastasis, the prognosis of GCC is poor, for 5-year overall survival (OS) rate is only about 9–25% [5–7]. Black and white ethnicity with GCC had a higher mortality rate than yellow ethnicity, and the eastern region had a better prognosis than the western region globally [8]. Given the prognosis of GCC was different from that of NGCC, it is necessary to explore this clinical issue further [9]. GCC doesn’t have specific symptoms at its early stages and lacks effective diagnostic techniques either, which might contribute to the increasing mortality rate [6, 10]. Studies have found that some clinical features were related to its poor prognosis. For example, it has been observed that tumor size and its anatomical location might be related to GCC outcome [11]. Therefore, it is encouraging and makes sense to predict the prognosis of GCC patients.

To help predict survival outcomes and make treatment decisions, the American Joint Committee on Cancer (AJCC) staging system has been developed and widely used to classify patients based on tumor, node, and metastasis (TNM) stage [12]. However, the AJCC staging system is still controversial to predict the prognosis of GCC patients who received comprehensive treatment [13]. In order to improve the accuracy of the survival estimations in GCC patients, a nomogram based on the traditional Cox proportional hazards (CPH) has been used to achieve that by some clinical researchers [14–17]. Nomogram is a graph that aggregates various predictive factors through multiple regression analysis, and can be used to intuitively predict patient outcomes, such as OS or cancer-specific survival rate (CSS). Nevertheless, these models had several limitations in time-to-event prediction for the clinical management [18]. CPH is conducted based on the linear hypotheses but the occurrence and development of tumors are influenced by many non-liner factors. So, it is not sufficient to perform linear hypotheses alone to explore the relationship between patients’ covariates (such as clinical and genetic characteristics) and the effectiveness of various treatment options in the real-world. Therefore, better models or methods are required for nonlinear variable analysis further.

With the rapid development of artificial intelligence (AI), AI is increasingly used in the various area. For example, Khan et al. [19] proposed a novel combination of optimized intelligent smart irrigation systems to improve the energy management performance of the system. Irshad et al. [20] developed a Heap Optimization Based Generalized Intelligent Neural Fuzzy Control (HO-GINFC) for estimating the cooling load of an air conditioning system with cold thermal storage. An artificial ecosystem optimization with Deep Learning Enabled Water Quality Prediction and Classification (AEODL-WQPC) model presented by Islam et al. [21] which was utilized to predict and categorize water quality level. Kumar et al. [22] established rooted elliptic curve cryptography with Vigenère cipher (RECC-VC) centered security amelioration on the IoMT to enhance security. Praveen et al. [23] found that the FastAI technology could be used with the ResNet-32 model to precisely identify breast ductal carcinoma. Vulli et al. [24] ascertained the fine-tuned DenseNet-169 had improved considerably histopathologic interpretation and diagnostic accuracy using the FastAI framework and the 1-cycle policy. Deep learning, also known as neural network, a research direction in the field of machine learning and AI, could be used to solve multifactor and nonlinear problems more appropriately [25]. Katzman et al. [26] developed a novel deep learning survival theory called DeepSurv, a multi-layer feed-forward network composed by an artificial neural network (ANN) and CPH, which could integrate the nonlinear risk function related to outcomes and was more flexible to deal with complex clinical factors in the real-world, so as to predict the result events. The authors and previous researchers have demonstrated that DeepSurv performed better than other linear prediction models like CPH and could be a useful tool in providing better treatment recommendations [27, 28].

Our study demonstrated GCC patients indeed have different survival time compared with NGCC patients. So we aimed to develop and validate a prognostic model for GCC patients by applying neural network survival theory DeepSurv, so that it might offer some references for doctors on clinical decision-making, after packaging it into a convenient windows desktop tool.

## Methods

### Research design and data sources

**Fig. 2:**
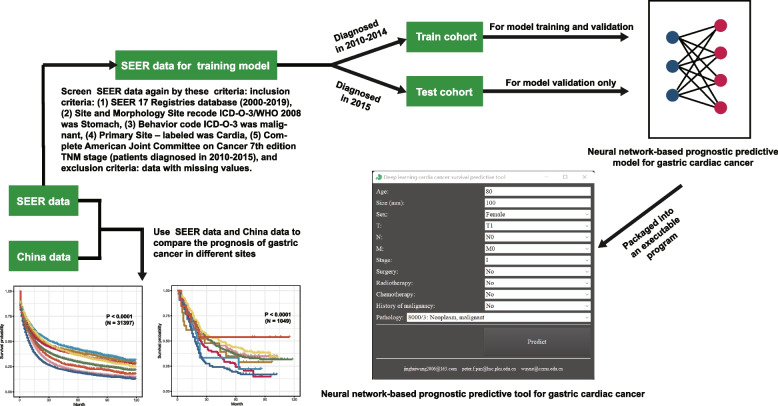
The flow chart of this study. SEER, the Surveillance, Epidemiology, and End Results database

This retrospective cohort study used clinical data from American the Surveillance, Epidemiology, and End Results (SEER) (https://seer.cancer.gov/) database and China National Human Genetic Resources Sharing Service Platform (http://www.superchip.com.cn/technology/Default.aspx). First, we compared the prognosis of GC occurring different sites using the American data and the Chinese data, following these criteria: patients were diagnosed with GC pathologically; complete tumor site record and follow-up information especially survival status; the SEER 17 Registries database (2000–2019) was used this time. After finding GCC might have different prognosis with NGCC, we screened SEER data again, to conduct GCC neural network-based predictive tool, following these inclusion criteria: (1) SEER 17 Registries database (2000–2019), (2) *Site and Morphology Site recode ICD-O-3/WHO 2008* was *Stomach*, (3) *Behavior code ICD-O-3* was *malignant*, (4) *Primary Site – labeled* was *Cardia*, (5) detailed AJCC 7^th^ edition TNM stage (patients diagnosed in 2010–2015), and following this exclusion criteria: data with missing values. As the Chinese data included many missing values on GCC patients’ therapy, they were not suitable to conduct the predictive tool. SEER data were then divided into two cohorts, the train cohort (patients diagnosed as GCC in 2010–2014) and the test cohort (diagnosed in 2015). The train cohort was utilized to conduct the neural network-based prognostic predictive model which validated by itself and the test cohort (Fig. 2). The principal study endpoint was OS. The follow-up cutoff date was December 31, 2019, according to the SEER research data description.

This study has been approved by the Ethics Committee of Beijing Chest Hospital affiliated to Capital Medical University (No. LW-2022–008).

### Predictive variables and pre-processing

According to clinical experience, age, sex, pathology, T, N, M, stage, size of tumor, surgery or not, radiotherapy or not, chemotherapy or not and history of malignancy were the predictive clinical features. According to the recording rules of SEER, age greater than 100 years old remained registered as 100. And survival time less than 1 month was regarded as 1. Before modeling, numerical clinical features (age and tumor size) were standardized (minus the mean divided by the standard deviation), and categorical variables were converted to dummy variables. Test cohort was processed in terms of the train cohort (Supplement Table 1).

### Neural network model training and packaging

To get more accurate prediction and avoid underfitting, we used batch normalization and batch training. Further, to avoid overfitting, early stopping callback and dropout layers were applied. The training curves were saved in Supplement Fig. 1.

Area under the receiver operating characteristics curve (AUC) was used to evaluate model performance. A better model usually scores an AUC closer to 1. As mentioned earlier, the model was trained using the train cohort, but evaluated with both the train and the test cohorts.

Finally, neural network-based prognostic predictive model for GCC was packaged into a tool (an executable program in Microsoft Windows 11 64bit).

### Statistical analysis

All statistical analyses were completed with R software (https://www.r-project.org). Wilcoxon signed-rank test was used for numerical data of skewed distribution, and Chi-square test was performed on categorical data. The Kaplan–Meier curves and log rank test were used to compare the prognosis of different tumor sites of stomach. *P* value of two-sided smaller than 0.05 was considered statistically significant.

## Results

### Prognosis of gastric cancer varies by sites

**Fig. 3:**
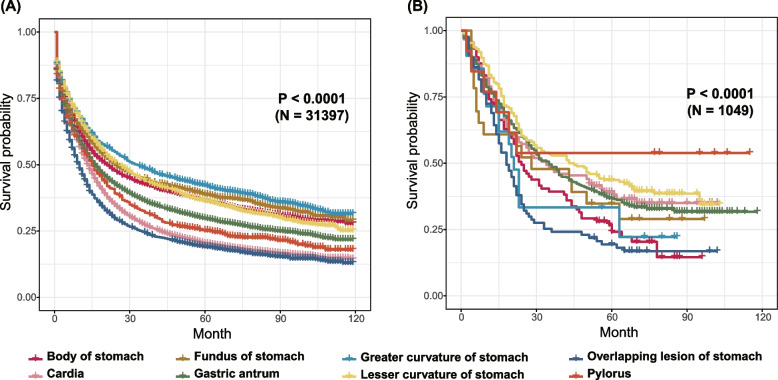
The prognosis of gastric cancer in different sites from: **A** the Surveillance, Epidemiology, and End Results database (SEER) data, (**B**) the Chinese data

We used the SEER data and the Chinese data to compare the prognosis of GC at the different sites. GCC might have different prognosis compared with other sites of GC. In SEER data, cancers in overlapping lesion of stomach had the worst prognosis, GCC the second worst and greater curvature of stomach had the best prognosis (*N* = 31,397, *P* < 0.0001). In China data, GC in overlapping lesion still had the worst survival, but pylorus cancer had the second worst prognosis and GCC had a moderate prognosis (*N* = 1049, *P* < 0.0001) (Fig. 3).

### Patients’ demographic information

Following our inclusion and exclusion criteria, 5371 patients were included finally. There were 4414 patients in train cohort and 957 patients in test cohort. As Table 1 showed, patients from both train cohort and test cohort had similar clinical features in age, sex, pathology, TNM stage, tumor size, surgery ratio, chemotherapy ratio and history of malignancy. The median age of train cohort was 67 years, and that of test cohort was the same. Train cohort included 20.80% females and test cohort had 21.11% females. The most common pathology was adenocarcinoma, in both cohorts. Most patients were staged T3, N0, or M0 in both cohorts. In train cohort, most patients were diagnosed as IV, but in test cohort IIIA was the most common stage. The median of tumor size was both 40 mm in two cohorts. In train and test cohorts, most patients got surgery or chemotherapy. But as for radiotherapy, test cohort patients most received it, while train cohort patients most not. And most patients in both two cohorts did not have a history of malignancy.

**Table 1:** Patients’ demographic information

	Train cohort	Test cohort	Statistical method	*P* value
	(2010–2014)	(2015)		
	(*N* = 4414)	(*N* = 957)		
	No. (%)			
Age			Wilcoxon signed-rank	0.4175
Median (IQR)	67 (58, 75)	67 (59, 74)		
Sex			Chi-square	0.8305
Female	918 (20.80)	202 (21.11)		
Male	3496 (79.20)	755 (78.89)		
Pathology			Chi-square	0.1929
8000/3: Neoplasm, malignant	7 (0.16)	2 (0.21)		
8010/3: Carcinoma, NOS	49 (1.11)	6 (0.63)		
8013/3: Large cell neuroendocrine carcinoma	5 (0.11)	3 (0.31)		
8020/3: Carcinoma, undifferentiated, NOS	4 (0.09)	2 (0.21)		
8021/3: Carcinoma, anaplastic, NOS	1 (0.02)	0 (0)		
8032/3: Spindle cell carcinoma, NOS	1 (0.02)	0 (0)		
8041/3: Small cell carcinoma, NOS	6 (0.14)	1 (0.10)		
8044/3: Small cell carcinoma, intermediate cell	0 (0)	1 (0.10)		
8045/3: Combined small cell carcinoma	2 (0.05)	0 (0)		
8046/3: Non-small cell carcinoma	2 (0.05)	0 (0)		
8051/3: Verrucous carcinoma, NOS	0 (0)	1 (0.10)		
8070/3: Squamous cell carcinoma, NOS	86 (1.95)	22 (2.30)		
8071/3: Squamous cell carcinoma, keratinizing, NOS	9 (0.20)	0 (0)		
8072/3: Squamous cell carcinoma, large cell, nonkeratinizing, NOS	2 (0.05)	0 (0)		
8140/3: Adenocarcinoma, NOS	3103 (70.30)	689 (72.00)		
8142/3: Linitis plastica	3 (0.07)	0 (0)		
8144/3: Adenocarcinoma, intestinal type	237 (5.37)	38 (3.97)		
8145/3: Carcinoma, diffuse type	50 (1.13)	12 (1.25)		
8210/3: Adenocarcinoma in adenomatous polyp	25 (0.57)	4 (0.42)		
8211/3: Tubular adenocarcinoma	21 (0.48)	1 (0.10)		
8240/3: Carcinoid tumor, NOS	24 (0.54)	11 (1.15)		
8244/3: Mixed adenoneuroendocrine carcinoma	2 (0.05)	1 (0.10)		
8246/3: Neuroendocrine carcinoma, NOS	33 (0.75)	10 (1.04)		
8255/3: Adenocarcinoma with mixed subtypes	69 (1.56)	15 (1.57)		
8260/3: Papillary adenocarcinoma, NOS	6 (0.14)	0 (0)		
8261/3: Adenocarcinoma in villous adenoma	1 (0.02)	0 (0)		
8263/3: Adenocarcinoma in tubulovillous adenoma	6 (0.14)	0 (0)		
8310/3: Clear cell adenocarcinoma, NOS	1 (0.02)	0 (0)		
8323/3: Mixed cell adenocarcinoma	2 (0.05)	0 (0)		
8480/3: Mucinous adenocarcinoma	79 (1.79)	16 (1.67)		
8481/3: Mucin-producing adenocarcinoma	23 (0.52)	3 (0.31)		
8490/3: Signet ring cell carcinoma	408 (9.24)	90 (9.40)		
8510/3: Medullary carcinoma, NOS	1 (0.02)	0 (0)		
8512/3: Medullary carcinoma with lymphoid stroma	1 (0.02)	1 (0.10)		
8560/3: Adenosquamous carcinoma	32 (0.72)	5 (0.52)		
8574/3: Adenocarcinoma with neuroendocrine differentiation	9 (0.20)	0 (0)		
8936/3: Gastrointestinal stromal sarcoma	104 (2.36)	22 (2.30)		
8980/3: Carcinosarcoma, NOS	0 (0)	1 (0.10)		
T			Chi-square	0.6922
T1	486 (11.01)	94 (9.82)		
T1a	421 (9.54)	102 (10.66)		
T1b	410 (9.29)	83 (8.67)		
T2	593 (13.43)	123 (12.85)		
T3	2091 (47.37)	464 (48.48)		
T4	54 (1.22)	12 (1.25)		
T4a	199 (4.51)	37 (3.87)		
T4b	160 (3.62)	42 (4.39)		
N			Chi-square	0.3275
N0	1928 (43.68)	436 (45.56)		
N1	1612 (36.52)	340 (35.53)		
N2	541 (12.26)	123 (12.85)		
N3	333 (7.54)	58 (6.06)		
M			Chi-square	0.3149
M0	3530 (79.97)	779 (81.40)		
M1	884 (20.03)	178 (18.60)		
Stage			Chi-square	0.1895
I	18 (0.41)	8 (0.84)		
IA	600 (13.59)	128 (13.38)		
IB	395 (8.95)	92 (9.61)		
II	18 (0.41)	0 (0)		
IIA	136 (3.08)	30 (3.13)		
IIB	759 (17.20)	170 (17.76)		
IIIA	849 (19.23)	190 (19.85)		
IIIB	342 (7.75)	87 (9.09)		
IIIC	412 (9.33)	74 (7.73)		
IV	885 (20.05)	178 (18.60)		
Size (mm)			Wilcoxon signed-rank	0.8093
Median (IQR)	40 (22, 55)	40 (20, 55)		
Surgery			Chi-square	0.1010
No	1810 (41.01)	420 (43.89)		
Yes	2604 (58.99)	537 (56.11)		
Radiotherapy			Chi-square	0.0069**
No	2242 (50.79)	440 (45.98)		
Yes	2172 (49.21)	517 (54.02)		
Chemotherapy			Chi-square	0.1061
No	1485 (33.64)	296 (30.93)		
Yes	2929 (66.36)	661 (69.07)		
History of malignancy			Chi-square	0.1407
No	3574 (80.97)	755 (78.89)		
Yes	840 (19.03)	202 (21.11)		

*IQR* Interquartile range, *NOS* Not otherwise specified

^**^P < 0.01

### Model performance and usage

**Fig. 4:**
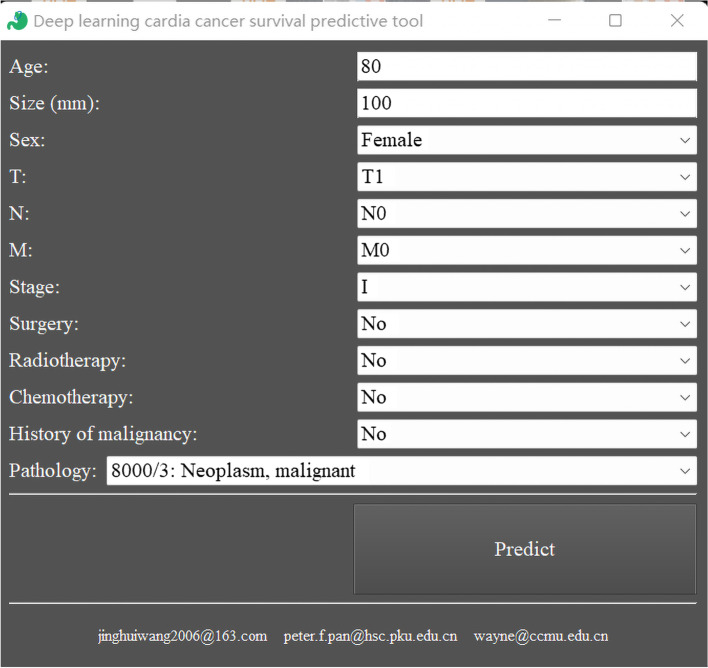
The interface of neural network-based prognostic predictive tool for gastric cardiac cancer

Neural network-based prognostic predictive model for GCC owned 0.7431 AUC in train cohort (95%, confidence intervals, CI, 0.7423–0.7439) and 0.7419 in test cohort (95% CI, 0.7411–0.7428) (Table 2). This model had a satisfactory performance. We then packaged it into an EXE file. When clicking the Main.exe file after unzipping Supplement File 1 (the linkage: https://drive.google.com/file/d/11-1k1rkx5fLuwcFAQuSlmqhtmVRTOt3q/view?usp=share_link), we could run the neural network-based prognostic predictive tool for GCC (Fig. 4). After clinician and researcher inputted a patient’s demographic information and clicked Predict button, the tool would calculate the OS possibility of this patient and draw his survival curves automatically. The survival curves were shown in users’ web browser, and could be zoomed in or out interactively to show the OS for a specific month.

**Table 2:** The performance of neural network-based prognostic predictive model for gastric cardiac cancer

	AUC	95% CI
Train cohort	0.7431	0.7423–0.7439
Test cohort	0.7419	0.7411–0.7428

*AUC* Area under the receiver operating characteristics curve, *CI* Confidence interval

## Discussion

GCC is a special malignant tumor located at the gastroesophageal junction (GEJ). The mucosa of gastric cardia is mainly composed of pure mucous and mixed mucoxyntic glands, with few parietal cells and scattered endocrine cells, but no chief cells [29]. It was reported that there were great differences from GCC with tumors of esophagus or distal stomach in epidemiological and biological behavior [6]. To date, its etiology is still unclear [30]. But some previous studies have shown that the prevalence of GCC was strongly correlated with aging, smoking, young women, Helicobacter pylori infection and Epstein-Barr virus (EBV) infection [31]. Meanwhile, there is no agreement on the accurate staging of GCC patients, though some studies have shown that GCC has a better prognosis than esophageal cancer when treated according to gastric cancer stages [32]. But some researchers did observe that the prognosis of GCC might be far worse than esophagus or other GC [33, 34]. So, to explore and compare the potential prognostic difference between GC and GCC, we used the Chinses data and SEER data to conduct survival analysis. As Kaplan–Meier curves illustrated, the prognosis of GCC patient in SEER database was worse than that of NGCC patients except for cancer from overlapping lesion of stomach, while it was not the second worst in the Chinese data, which was similar to previous studies [8, 35]. Some researchers reported that these differences might be related to the surgical method and the number of lymph node resection [36, 37], but more studies are still required. Thus, as far as we have found so far, it might need to treat GC and GCC differently.

To date, surgical resection has still been the most important treatment for early GCC patients [37]. For patients who were not suitable for gastrectomy, endoscopic submucosal dissection (ESD) resection could also achieve a good prognosis because of the low rate of lymph node metastasis in early GCC [38, 39]. Long et al. [10] found that the increased lymph node removal and chemoradiotherapy (CRT) contributed to improving the survival rate of GCC patients. And various other risk factors affecting the prognosis of these patients had been reported too, including sex, age, smoking, alcohol, histological type and TNM stage [40–42]. Therefore, it is necessary and feasible to develop a survival model to predict the prognosis of GCC patients based on clinical features. Previous studies have reported a few models to predict the survival of GCC patients [14–17]. For example, Shi et al. [14] demonstrated that a CPH-based nomogram’s consistency index (C-index) was 0.714 (95% CI, 0.705–0.723) and 0.734 (95% CI, 0.721–0.747) in training cohort and validation cohort, respectively when predicting GCC patients’ OS. Likewise, Chen et al. [15] built a CPH-based nomogram with only 0.590 (95% CI, 0.569–0.611) C-index in the training cohort and 0.569 (95% CI, 0.532–0.606) in the validation cohort when predicting GCC OS. Few parameters were incorporated in these studies, and the latter focused only on the prognosis of metastatic cancer, these might be the cause of the model's weak performance. Similarly, Liu et al. [16] created a CPH nomogram with 0.726 calibration index, and the model was established in one cohort only and not validated externally (Supplement Table 2). Obviously, all of these models behaved generally and had their own defects. They assumed that the risk of death was a simple linear combination of its covariates, which might be too idealistic in a real clinical world. Therefore, the prediction accuracy of these models has been limited, and developing a more reasonable survival prediction model which incorporates nonlinear factors has become an exploration direction to researchers.

As we know, deep learning models have been widely used in the diagnosis of endoscopic and histopathology of GC [7, 43–46], evaluation of tumor invasion depth and lymph node metastasis [47–49] and the prediction of treatment efficacy [50, 51]. These deep learning models have shown satisfactory performance in their respective fields. Excitingly, a novel deep learning theory called DeepSurv developed by Katzman et al. [26] in 2018, which combined deep learning with ANN and CPH, has achieved initial success in the survival prediction of some cancers. For example, She et al. [52] found that DeepSurv model was significantly better than the traditional AJCC TNM staging system in non-small-cell lung-cancer-specific survival (C-index = 0.739 vs 0.706). Huang et al. [53] demonstrated that the DeepSurv model was superior to the TNM staging model in predicting esophageal CSS with the internal test dataset (C-index = 0.753 vs 0.638) and external validation dataset (C-index = 0.687 vs 0.643). These suggested that the deep learning neural network model could be more widely used as a potential tool to assist clinicians with prognosis prediction. To our knowledge, there was no study using deep learning models to predict survival in patients with GCC.

In this study, the deep learning algorithm was used to analyze the large-scale GCC clinical data and conduct a neural network tool for the first time. The AUC was 0.7431 (95% CI, 0.7423–0.7439) for the train cohort and 0.7419 (95% CI, 0.7411–0.7428) for the test cohort when applying this prediction model. These results showed that this model might have more advantages than previous models in predicting the OS of GCC patients. Finally, we converted the model into a desktop tool to use conveniently, hoping it could offer some references for clinicians and researchers (Supplement File 1).

## Limitations

Some characteristic information of GCC patients from SEER database was incomplete, such as surgical methods, chemotherapy types and tumor markers, which might be important to GC patients’ prognosis. And large-scale prospective multicenter data was needed for further verification.

## Conclusions

GCC patients indeed have different survival time compared with non GCC patients. And the neural network-based prognostic predictive tool developed in this study is a novel and promising software for the clinical outcome analysis of GCC patients.

## Supplementary Information

**Additional file 1: Supplement Figure 1.** The training curves of neural network-based prognostic predictive model for GCC.

**Additional file 2: Supplement File 1.** The Neural network-based prognostic predictive tool for GCC patients.

**Additional file 3: Supplement File 2.** The raw data of this study.

**Additional file 4: Supplement Table 1.** The mean and standard deviation of numerical clinical features in train cohort.

**Additional file 5: Supplement Table 2.** The models’ performance of previous research in overall survival.

## Data Availability

The neural network-based prognostic predictive tool for GCC has been uploaded, saved in Supplement File 1. The raw data were saved in Supplement File 2.

## Abbreviations

GC: Gastric cancer
GCC: Gastric cardiac cancer
NGCC: Non-cardiac gastric cancer
OS: Overall survival
AJCC: American Joint Committee on Cancer
TNM: Tumor, node, and metastasis
CPH: Cox proportional hazards
CSS: Cancer-specific survival
AI: Artificial intelligence
HO-GINFC: Heap Optimization Based Generalized Intelligent Neural Fuzzy Control
RECC-VC: Rooted elliptic curve cryptography with Vigenère cipher
ANN: Artificial neural network
SEER: The Surveillance, Epidemiology, and End Results database
AUC: Area under the receiver operating characteristics curve
CI: Confidence intervals
GEJ: Gastroesophageal junction
EBV: Epstein-Barr virus
ESD: Endoscopic submucosal dissection
CRT: Chemoradiotherapy
C-index: Consistency index
