# Supplementary figures and images for: Neural network-based prognostic predictive tool for gastric cardiac cancer: the worldwide retrospective study
Source: BioData Min. 2023 Jul 18;16:21. doi: 10.1186/s13040-023-00335-z (PMC10353146; doi:10.1186/s13040-023-00335-z)

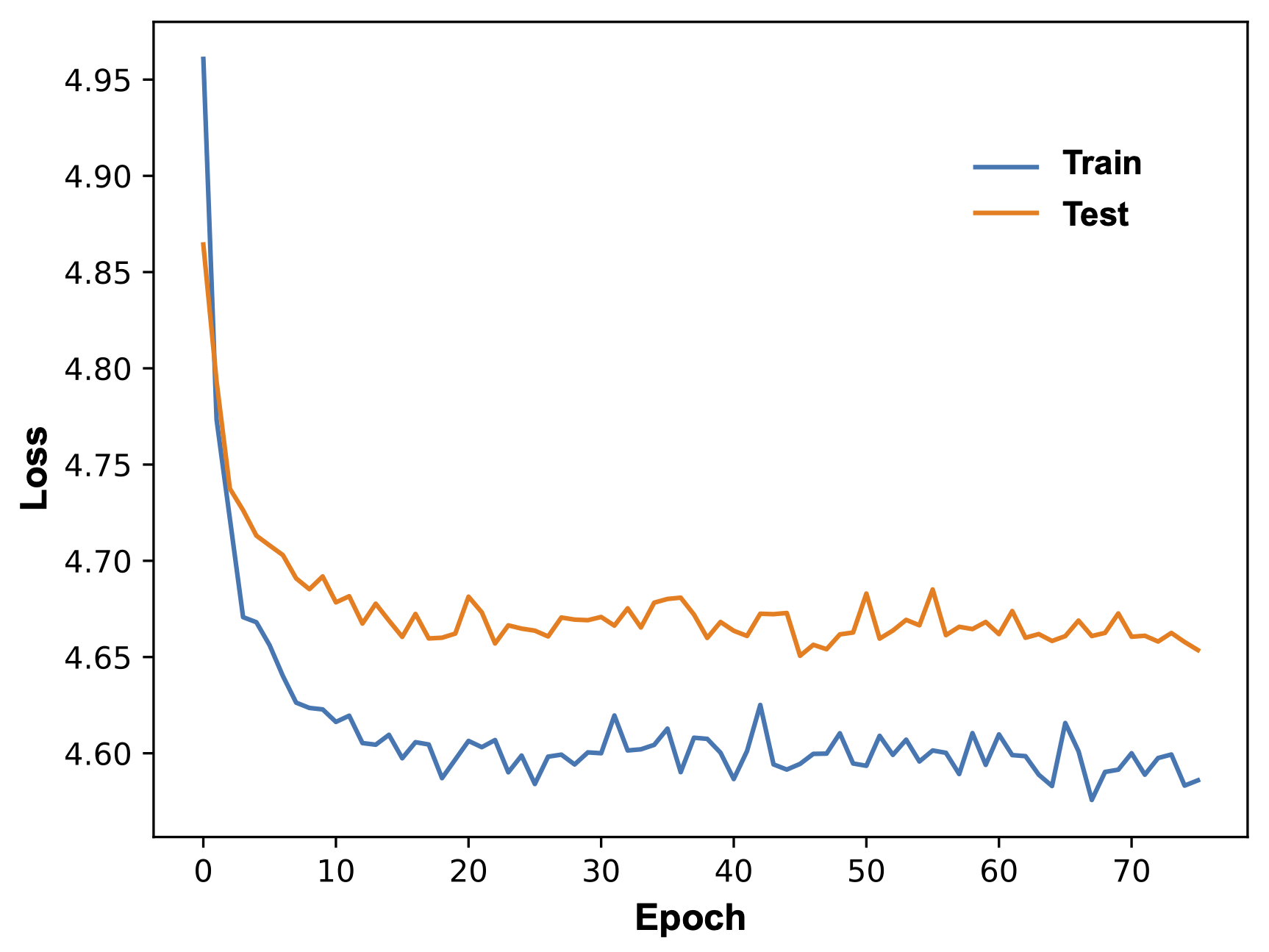

Supplement: Supplementary file 1 — Additional file 1: Supplement Figure 1. The training curves of neural network-based prognostic predictive model for GCC. [file 13040_2023_335_MOESM1_ESM.tiff]

Supplement: Supplementary file 2 — Additional file 2: Supplement File 1. The Neural network-based prognostic predictive tool for GCC patients. [file 13040_2023_335_MOESM2_ESM.zip › Supplement File 1/IPython/core/tests/2x2.png]

Supplement: Supplementary file 2 — Additional file 2: Supplement File 1. The Neural network-based prognostic predictive tool for GCC patients. [file 13040_2023_335_MOESM2_ESM.zip › Supplement File 1/IPython/core/tests/2x2.jpg]
