# Supplementary material for: Neural network-based prognostic predictive tool for gastric cardiac cancer: the worldwide retrospective study
Source: BioData Min. 2023 Jul 18;16:21. doi: 10.1186/s13040-023-00335-z (PMC10353146; doi:10.1186/s13040-023-00335-z)
Supplement: Supplementary file 4 — Additional file 4: Supplement Table 1. The mean and standard deviation of numerical clinical features in train cohort. [file 13040_2023_335_MOESM4_ESM.docx]

| **Supplement Table 1** The mean and standard deviation of numerical clinical features in train cohort. | | |
| --- | --- | --- |
|  | Age (year) | Size (mm) |
| Mean | 66.16 | 43.02 |
| Standard deviation | 12.12 | 36.02 |
