# Supplementary material for: Neural network-based prognostic predictive tool for gastric cardiac cancer: the worldwide retrospective study
Source: BioData Min. 2023 Jul 18;16:21. doi: 10.1186/s13040-023-00335-z (PMC10353146; doi:10.1186/s13040-023-00335-z)
Supplement: Supplementary file 5 — Additional file 5: Supplement Table 2. The models’ performance of previous research in overall survival. [file 13040_2023_335_MOESM5_ESM.docx]

| Supplement Table 2 The models’ performance of previous research in overall survival. | | | | | | |
| --- | --- | --- | --- | --- | --- | --- |
| Study | Predictive factors | Total patients | Train cohort | Test cohort | AUC/C-index | |
|  | Number | | | | Train cohort | Test cohort |
| Shi et al | 10 | 7332 | 5231 | 2200 | 0.714 (95% CI, 0.705-0.723) | 0.734 (95% CI, 0.721-0.747) |
| Chen et al | 7 | 1616 | 1212 | 404 | 0.590 (95% CI, 0.569-0.611) | 0.569 (95% CI, 0.532-0.606) |
| Liu et al | 6 | 8013 | / | / | 0.726 | / |
| This study | 12 | 5371 | 4414 | 957 | 0.7431 (95% CI, 0.7423-0.7439) | 0.7419 (95% CI, 0.7411-0.7428) |
| AUC, area under the receiver operating characteristics curve. C-index, consistency index. CI, confidence interval. (The AUC and C exponents are usually mixed when the dependent variables are binary variables). | | | | | | |
