# Supplementary material for: Neural network-based prognostic predictive tool for gastric cardiac cancer: the worldwide retrospective study
Source: BioData Min. 2023 Jul 18;16:21. doi: 10.1186/s13040-023-00335-z (PMC10353146; doi:10.1186/s13040-023-00335-z)
Supplement: Supplementary file 2 — Additional file 2: Supplement File 1. The Neural network-based prognostic predictive tool for GCC patients. [file 13040_2023_335_MOESM2_ESM.zip › Supplement File 1/notebook/templates/tree.html]

{% extends "page.html" %}
{% block title %}{{page\_title}}{% endblock %}
{% block params %}
{{super()}}
data-base-url="{{base\_url | urlencode}}"
data-notebook-path="{{notebook\_path | urlencode}}"
data-terminals-available="{{terminals\_available}}"
data-server-root="{{server\_root}}"
{% endblock %}
{% block headercontainer %}
{{super()}}
{% if shutdown\_button %}

{% trans %}Quit{% endtrans %}
{% endif %}
{% endblock %}
{% block site %}

- {% trans %}Files{% endtrans %}
- {% trans %}Running{% endtrans %}
- {% trans %}Clusters{% endtrans %}

{% trans %}Select items to perform actions on them.{% endtrans %}

{% trans %}Duplicate{% endtrans %} 

{% trans %}Duplicate selected{% endtrans %}

{% trans %}Rename{% endtrans %}

{% trans %}Rename selected{% endtrans %}

{% trans %}Move{% endtrans %}

{% trans %}Move selected{% endtrans %}

{% trans %}Download{% endtrans %}

{% trans %}Download selected{% endtrans %}

{% trans %}Shutdown{% endtrans %}

{% trans %}Shutdown selected notebook(s){% endtrans %}

{% trans %}View{% endtrans %}

{% trans %}View selected{% endtrans %}

{% trans %}Edit{% endtrans %}

{% trans %}Edit selected{% endtrans %}


{% trans %}Delete selected{% endtrans %}

{% trans %}Upload{% endtrans %}

{% trans %}New{% endtrans %}

{% trans %}Toggle Dropdown{% endtrans %}

- Notebook:
- Other:
- {% trans %}Text File{% endtrans %}
- {% trans %}Folder{% endtrans %}
{% if terminals\_available %}- {% trans %}Terminal{% endtrans %}
{% else %}- {% trans %}Terminals Unavailable{% endtrans %}
{% endif %}

checkbox

Toggle Dropdown

- {% trans %}Folders{% endtrans %}
- {% trans %}All Notebooks{% endtrans %}
- {% trans %}Running{% endtrans %}
- {% trans %}Files{% endtrans %}

{% for crumb in breadcrumbs[1:] %}- {{crumb[1]}}
{% endfor %}

{% trans %}Name{% endtrans %}

{% trans %}Last Modified{% endtrans %}

{% trans %}File size{% endtrans %}

{% trans %}Currently running Jupyter processes{% endtrans %}

Terminals

{% if terminals\_available %}

{% trans %}There are no terminals running.{% endtrans %}

{% else %}

{% trans %}Terminals are unavailable.{% endtrans %}

{% endif %}

{% trans %}Notebooks{% endtrans %}

{% trans %}There are no notebooks running.{% endtrans %}

{% trans %}Clusters tab is now provided by IPython parallel.{% endtrans %}
{% trans %}See 'IPython parallel' for installation details.{% endtrans %}

{% endblock %}
{% block script %}
{{super()}}
{% endblock %}
