# Supplementary material for: Neural network-based prognostic predictive tool for gastric cardiac cancer: the worldwide retrospective study
Source: BioData Min. 2023 Jul 18;16:21. doi: 10.1186/s13040-023-00335-z (PMC10353146; doi:10.1186/s13040-023-00335-z)
Supplement: Supplementary file 2 — Additional file 2: Supplement File 1. The Neural network-based prognostic predictive tool for GCC patients. [file 13040_2023_335_MOESM2_ESM.zip › Supplement File 1/notebook/templates/page.html]

{% block title %}Jupyter Notebook{% endblock %}
{% block favicon %}{% endblock %}

{% block stylesheet %}
{% endblock %}
{% block meta %}
{% endblock %}


{% trans %}Jupyter Notebook requires JavaScript.{% endtrans %}  
{% trans %}Please enable it to proceed. {% endtrans %}

{% block logo %}{% endblock %}

{% block headercontainer %}
{% endblock %}
{% block header\_buttons %}
{% block login\_widget %}
{% if logged\_in %}
{% trans %}Logout{% endtrans %}
{% elif login\_available and not logged\_in %}
{% trans %}Login{% endtrans %}
{% endif %}
{% endblock %}
{% endblock header\_buttons %}

{% block header %}
{% endblock %}

{% block site %}
{% endblock %}

{% block after\_site %}
{% endblock %}
{% block script %}
{% endblock %}
