# Supplementary material for: Neural network-based prognostic predictive tool for gastric cardiac cancer: the worldwide retrospective study
Source: BioData Min. 2023 Jul 18;16:21. doi: 10.1186/s13040-023-00335-z (PMC10353146; doi:10.1186/s13040-023-00335-z)
Supplement: Supplementary file 2 — Additional file 2: Supplement File 1. The Neural network-based prognostic predictive tool for GCC patients. [file 13040_2023_335_MOESM2_ESM.zip › Supplement File 1/notebook/templates/terminal.html]

{% extends "page.html" %}
{% block title %}{{page\_title}}{% endblock %}
{% block favicon %}{% endblock %}
{% block bodyclasses %}terminal-app {{super()}}{% endblock %}
{% block params %}
data-base-url="{{base\_url | urlencode}}"
data-ws-url="{{ws\_url | urlencode}}"
data-ws-path="{{ws\_path}}"
{% endblock %}
{% block stylesheet %}
{{super()}}

{% endblock %}
{% block headercontainer %}
{{super()}}
{% endblock headercontainer %}
{% block site %}

{% endblock %}
{% block script %}
{{super()}}
{% endblock %}
