# Supplementary material for: Neural network-based prognostic predictive tool for gastric cardiac cancer: the worldwide retrospective study
Source: BioData Min. 2023 Jul 18;16:21. doi: 10.1186/s13040-023-00335-z (PMC10353146; doi:10.1186/s13040-023-00335-z)
Supplement: Supplementary file 2 — Additional file 2: Supplement File 1. The Neural network-based prognostic predictive tool for GCC patients. [file 13040_2023_335_MOESM2_ESM.zip › Supplement File 1/notebook/templates/login.html]

{% extends "page.html" %}
{% block stylesheet %}
{{super()}}
{% endblock %}
{% block login\_widget %}
{% endblock %}
{% block site %}

{% if login\_available %}
{# login\_available means password-login is allowed. Show the form. #}

{{ xsrf\_form\_html() | safe }}
{% if token\_available %}
**{% trans %}Password or token:{% endtrans %}**
{% else %}
**{% trans %}Password:{% endtrans %}**
{% endif %}
{% trans %}Log in{% endtrans %}

{% else %}

{% trans %}No login available, you shouldn't be seeing this page.{% endtrans %}

{% endif %}
{% if message %}

{% for key in message %}

{{message[key]}}

{% endfor %}

{% endif %}
{% if token\_available %}
{% block token\_message %}

### Token authentication is enabled

If no password has been configured, you need to open the notebook
server with its login token in the URL, or paste it above.
This requirement will be lifted if you
 **enable a password**.

The command:

```
jupyter notebook list
```

will show you the URLs of running servers with their tokens,
which you can copy and paste into your browser. For example:

```
Currently running servers:
http://localhost:8888/?token=c8de56fa... :: /Users/you/notebooks
```

or you can paste just the token value into the password field on this
page.

See
 **the documentation on how to enable a password** 
in place of token authentication,
if you would like to avoid dealing with random tokens.

Cookies are required for authenticated access to notebooks.

{% if allow\_password\_change %}

### {% trans %}Setup a Password{% endtrans %}

You can also setup a password by entering your token and a new password
on the fields below:

{{ xsrf\_form\_html() | safe }}

#### Token

#### New Password

{% trans %}Log in and set new password{% endtrans %}

{% endif %}

{% endblock token\_message %}
{% endif %}

{% endblock %}
{% block script %}
{{super()}}
{% endblock %}
