# Supplementary material for: Neural network-based prognostic predictive tool for gastric cardiac cancer: the worldwide retrospective study
Source: BioData Min. 2023 Jul 18;16:21. doi: 10.1186/s13040-023-00335-z (PMC10353146; doi:10.1186/s13040-023-00335-z)
Supplement: Supplementary file 2 — Additional file 2: Supplement File 1. The Neural network-based prognostic predictive tool for GCC patients. [file 13040_2023_335_MOESM2_ESM.zip › Supplement File 1/notebook/templates/browser-open.html]

{# This template is not served, but written as a file to open in the browser,
passing the token without putting it in a command-line argument. #}


Opening Jupyter Notebook


This page should redirect you to Jupyter Notebook. If it doesn't,
click here to go to Jupyter.
