# Supplementary material for: Neural network-based prognostic predictive tool for gastric cardiac cancer: the worldwide retrospective study
Source: BioData Min. 2023 Jul 18;16:21. doi: 10.1186/s13040-023-00335-z (PMC10353146; doi:10.1186/s13040-023-00335-z)
Supplement: Supplementary file 2 — Additional file 2: Supplement File 1. The Neural network-based prognostic predictive tool for GCC patients. [file 13040_2023_335_MOESM2_ESM.zip › Supplement File 1/notebook/templates/notebook.html]

{% extends "page.html" %}
{% block favicon %}{% endblock %}
{% block stylesheet %}
{% if mathjax\_url %}
{% endif %}

{{super()}}

{% endblock %}
{% block bodyclasses %}notebook\_app {{super()}}{% endblock %}
{% block params %}
{{super()}}
data-base-url="{{base\_url | urlencode}}"
data-ws-url="{{ws\_url | urlencode}}"
data-notebook-name="{{notebook\_name | urlencode}}"
data-notebook-path="{{notebook\_path | urlencode}}"
{% endblock %}
{% block headercontainer %}


{{super()}}
{% block kernel\_logo\_widget %}
{% endblock %}
{% endblock headercontainer %}
{% block header %}

{% trans %}Menu{% endtrans %}

{% trans %}Kernel{% endtrans %}

 


- {% trans %}File{% endtrans %}
  - {% trans %}New Notebook{% endtrans %}Dropdown
  - {% trans %}Open...{% endtrans %}
  - {% trans %}Make a Copy...{% endtrans %}
  - {% trans %}Save as...{% endtrans %}
  - {% trans %}Rename...{% endtrans %}
  - {% trans %}Save and Checkpoint{% endtrans %}
  - {% trans %}Revert to Checkpoint{% endtrans %}Dropdown
  - {% trans %}Print Preview{% endtrans %}
  - {% trans %}Download as{% endtrans %}Dropdown
    {% for exporter in get\_frontend\_exporters() %}- {{ exporter.display }}
    {% endfor %}
  - {% trans %}Deploy as{% endtrans %}
  - {% trans %}Trust Notebook{% endtrans %}
  - {% trans %}Close and Halt{% endtrans %}
- {% trans %}Edit{% endtrans %}
  - {% trans %}Cut Cells{% endtrans %}
  - {% trans %}Copy Cells{% endtrans %}
  - {% trans %}Paste Cells Above{% endtrans %}
  - {% trans %}Paste Cells Below{% endtrans %}
  - {% trans %}Paste Cells & Replace{% endtrans %}
  - {% trans %}Delete Cells{% endtrans %}
  - {% trans %}Undo Delete Cells{% endtrans %}
  - {% trans %}Split Cell{% endtrans %}
  - {% trans %}Merge Cell Above{% endtrans %}
  - {% trans %}Merge Cell Below{% endtrans %}
  - {% trans %}Move Cell Up{% endtrans %}
  - {% trans %}Move Cell Down{% endtrans %}
  - {% trans %}Edit Notebook Metadata{% endtrans %}
  - {% trans %}Find and Replace{% endtrans %}
  - {% trans %}Cut Cell Attachments{% endtrans %}
  - {% trans %}Copy Cell Attachments{% endtrans %}
  - {% trans %}Paste Cell Attachments{% endtrans %}
  - {% trans %}Insert Image{% endtrans %}
- {% trans %}View{% endtrans %}
  - {% trans %}Toggle Header{% endtrans %}
  - {% trans %}Toggle Toolbar{% endtrans %}
  - {% trans %}Toggle Line Numbers{% endtrans %}
  - {% trans %}Cell Toolbar{% endtrans %}
- {% trans %}Insert{% endtrans %}
  - {% trans %}Insert Cell Above{% endtrans %}
  - {% trans %}Insert Cell Below{% endtrans %}
- {% trans %}Cell{% endtrans %}
  - {% trans %}Run Cells{% endtrans %}
  - {% trans %}Run Cells and Select Below{% endtrans %}
  - {% trans %}Run Cells and Insert Below{% endtrans %}
  - {% trans %}Run All{% endtrans %}
  - {% trans %}Run All Above{% endtrans %}
  - {% trans %}Run All Below{% endtrans %}
  - {% trans %}Cell Type{% endtrans %}
    - Code
    - {% trans %}Markdown{% endtrans %}
    - {% trans %}Raw NBConvert{% endtrans %}
  - {% trans %}Current Outputs{% endtrans %}
    - {% trans %}Toggle{% endtrans %}
    - {% trans %}Toggle Scrolling{% endtrans %}
    - {% trans %}Clear{% endtrans %}
  - {% trans %}All Output{% endtrans %}
    - {% trans %}Toggle{% endtrans %}
    - {% trans %}Toggle Scrolling{% endtrans %}
    - {% trans %}Clear{% endtrans %}
- {% trans %}Kernel{% endtrans %}
  - {% trans %}Interrupt{% endtrans %}
  - {% trans %}Restart{% endtrans %}
  - {% trans %}Restart & Clear Output{% endtrans %}
  - {% trans %}Restart & Run All{% endtrans %}
  - {% trans %}Reconnect{% endtrans %}
  - {% trans %}Shutdown{% endtrans %}
  - {% trans %}Change kernel{% endtrans %}
- {% trans %}Help{% endtrans %}
  {% block help %}- {% trans %}User Interface Tour{% endtrans %}
  - {% trans %}Keyboard Shortcuts{% endtrans %}
  - {% trans %}Edit Keyboard Shortcuts{% endtrans %}
  {% set
  sections = (
  (
  ("http://nbviewer.jupyter.org/github/ipython/ipython/blob/3.x/examples/Notebook/Index.ipynb", \_("Notebook Help"), True),
  ("https://help.github.com/articles/markdown-basics/",\_("Markdown"),True),
  ),
  )
  %}
  {% set openmsg = \_("Opens in a new window") %}
  {% for helplinks in sections %}
  {% for link in helplinks %}- {% if link[2] %}
    {% endif %}
    {{link[1]}}
  {% endfor %}
  {% if not loop.last %}
  {% endif %}
  {% endfor %}
  - {% trans %}About{% endtrans %}
  {% endblock %}

{% endblock header %}
{% block site %}

{% endblock %}
{% block after\_site %}

{% endblock %}
{% block script %}
{{super()}}
{% endblock %}
