# Supplementary material for: Neural network-based prognostic predictive tool for gastric cardiac cancer: the worldwide retrospective study
Source: BioData Min. 2023 Jul 18;16:21. doi: 10.1186/s13040-023-00335-z (PMC10353146; doi:10.1186/s13040-023-00335-z)
Supplement: Supplementary file 2 — Additional file 2: Supplement File 1. The Neural network-based prognostic predictive tool for GCC patients. [file 13040_2023_335_MOESM2_ESM.zip › Supplement File 1/notebook/templates/edit.html]

{% extends "page.html" %}
{% block title %}{{page\_title}}{% endblock %}
{% block favicon %}{% endblock %}
{% block stylesheet %}

{{super()}}
{% endblock %}
{% block bodyclasses %}edit\_app {{super()}}{% endblock %}
{% block params %}
data-base-url="{{base\_url | urlencode}}"
data-file-path="{{file\_path}}"
{{super()}}
{% endblock %}
{% block headercontainer %}


{{super()}}
{% endblock %}
{% block header %}

{% trans %}current mode{% endtrans %}

Menu


- {% trans %}File{% endtrans %}
  - {% trans %}New{% endtrans %}
  - {% trans %}Save{% endtrans %}
  - {% trans %}Rename{% endtrans %}
  - {% trans %}Download{% endtrans %}
- {% trans %}Edit{% endtrans %}
  - {% trans %}Find{% endtrans %}
  - {% trans %}Find & Replace{% endtrans %}
  - {% trans %}Key Map{% endtrans %}
  - {% trans %}Default{% endtrans %}
  - {% trans %}Sublime Text{% endtrans %}
  - Vim
  - emacs
- {% trans %}View{% endtrans %}
  - {% trans %}Toggle Header{% endtrans %}
  - {% trans %}Toggle Line Numbers{% endtrans %}
- {% trans %}Language{% endtrans %}

{% endblock %}
{% block site %}

{% endblock %}
{% block script %}
{{super()}}
{% endblock %}
