# Supplementary material for: Neural network-based prognostic predictive tool for gastric cardiac cancer: the worldwide retrospective study
Source: BioData Min. 2023 Jul 18;16:21. doi: 10.1186/s13040-023-00335-z (PMC10353146; doi:10.1186/s13040-023-00335-z)
Supplement: Supplementary file 2 — Additional file 2: Supplement File 1. The Neural network-based prognostic predictive tool for GCC patients. [file 13040_2023_335_MOESM2_ESM.zip › Supplement File 1/notebook/templates/logout.html]

{% extends "page.html" %}
{# This template is rendered in response to an authenticated request, so the
user is technically logged in. But when the user sees it, the cookie is
cleared by the Javascript, so we should render this as if the user was logged
out, without e.g. authentication tokens.
#}
{% set logged\_in = False %}
{% block stylesheet %}
{{super()}}
{% endblock %}
{% block login\_widget %}
{% endblock %}
{% block site %}

{% if message %}
{% for key in message %}

{{message[key]}}

{% endfor %}
{% endif %}
{% if not login\_available %}
{% trans %}Proceed to the dashboard{% endtrans %}.
{% else %}
{% trans %}Proceed to the login page{% endtrans %}.
{% endif %}

{% endblock %}
{% block script %}
{{super()}}
{% endblock %}
