# Supplementary material for: Neural network-based prognostic predictive tool for gastric cardiac cancer: the worldwide retrospective study
Source: BioData Min. 2023 Jul 18;16:21. doi: 10.1186/s13040-023-00335-z (PMC10353146; doi:10.1186/s13040-023-00335-z)
Supplement: Supplementary file 2 — Additional file 2: Supplement File 1. The Neural network-based prognostic predictive tool for GCC patients. [file 13040_2023_335_MOESM2_ESM.zip › Supplement File 1/notebook/templates/error.html]

{% extends "page.html" %}
{% block login\_widget %}
{% endblock %}
{% block stylesheet %}
{{super()}}
{% endblock %}
{% block site %}

{% block h1\_error %}

# {{status\_code}} : {{status\_message}}

{% endblock h1\_error %}
{% block error\_detail %}
{% if message %}

{% trans %}The error was:{% endtrans %}

```
{{message}}
```

{% endif %}
{% endblock %}

{% endblock %}
{% block script %}
{{super()}}
{% endblock script %}
